# Maize-IAS: a maize image analysis software using deep learning for high-throughput plant phenotyping

**DOI:** 10.1186/s13007-021-00747-0

**Published:** 2021-04-29

**Authors:** Shuo Zhou, Xiujuan Chai, Zixuan Yang, Hongwu Wang, Chenxue Yang, Tan Sun

**Affiliations:** 1 Agricultural Information Institute, Chinese Academy of Agricultural Sciences, No.12 Zhongguancun South St., Beijing, 100081 China; 2 Institute of Crop Sciences, Chinese Academy of Agricultural Sciences, No.12 Zhongguancun South St., Beijing, 100081 China; 3 Key Laboratory of Big Agri-Data, Ministry of Agriculture, Beijing, China

**Keywords:** Maize phenotyping, Instance segmentation, Computer vision, Deep learning, Convolutional neural network

## Abstract

**Background:**

Maize (Zea mays L.) is one of the most important food sources in the world and has been one of the main targets of plant genetics and phenotypic research for centuries. Observation and analysis of various morphological phenotypic traits during maize growth are essential for genetic and breeding study. The generally huge number of samples produce an enormous amount of high-resolution image data. While high throughput plant phenotyping platforms are increasingly used in maize breeding trials, there is a reasonable need for software tools that can automatically identify visual phenotypic features of maize plants and implement batch processing on image datasets.

**Results:**

On the boundary between computer vision and plant science, we utilize advanced deep learning methods based on convolutional neural networks to empower the workflow of maize phenotyping analysis. This paper presents Maize-IAS (Maize Image Analysis Software), an integrated application supporting one-click analysis of maize phenotype, embedding multiple functions: (I) Projection, (II) Color Analysis, (III) Internode length, (IV) Height, (V) Stem Diameter and (VI) Leaves Counting. Taking the RGB image of maize as input, the software provides a user-friendly graphical interaction interface and rapid calculation of multiple important phenotypic characteristics, including leaf sheath points detection and leaves segmentation. In function Leaves Counting, the mean and standard deviation of difference between prediction and ground truth are 1.60 and 1.625.

**Conclusion:**

The Maize-IAS is easy-to-use and demands neither professional knowledge of computer vision nor deep learning. All functions for batch processing are incorporated, enabling automated and labor-reduced tasks of recording, measurement and quantitative analysis of maize growth traits on a large dataset. We prove the efficiency and potential capability of our techniques and software to image-based plant research, which also demonstrates the feasibility and capability of AI technology implemented in agriculture and plant science.

## Background

Multiple phenotypic traits constantly change over time in the vegetative stage of the life cycle of maize (Zea mays L.), reflecting the growth status of maize and are popularly used by plant researchers to evaluate the impact of specifically defined treatments and experimental variables on maize [1–3]. As advanced Non-invasive and high throughput plant phenotyping platforms (HTPPs) provide the possibility to automatically monitor and record dynamics morphological traits of maize plants in a large scale of cultivation, booming data volume makes processing them an urgent problem.

In recent years, research progress of image-based plant phenotyping have been made [4–7]. A range of hardware and software solutions are developed aiming at some specific traits with different levels of automation and throughput. On the field scene, unmanned aerial platform (UAP) shows its potential to rapidly and cost-effectively phenotype large numbers of plots by time series: M Zaman-Allah uses a UAP equipped with sensors [8] for multi-spectral imaging for low-nitrogen stress tolerance in maize. Liebisch et al. proposes a method [9] for remote phenotyping of maize genotypes using the Zeppelin NT aircraft, which has the ability of monitoring throughout the season, robust image segmentation and the identification of individual plots in images. An UAV-assisted HTPP framework [10] is used for preselecting maize phenotypic components. In laboratories scene, software systems have been assisting researchers to quickly quantify traits of interest: T.E.Grift presents a measurement system [11] consisted of a semi-automated imaging box that provided a highly diffuse lighting scene and allowing imaging of up 700 roots per day. TIPS [7] enables morphological features of maize tassels to be quantified automatically at a scale that supports population-level studies. Nocolas Brichet presents a pipeline [12] combining computer vision, machine learning, and robotics, which tracks the growth of maize ear and silks and applies large-scale genetic analyses in a non-invasive and automatized way.

Region of interest extraction, namely plant region segmentation, is the primary function provided by software and papers mentioned above. ImageJ [13], PlantCV [14], HTPhone [15] and Image Harvest [16], like most of other open-source plant image processing software and libraries published before, mostly utilize digital image processing algorithms for their main functions. To extract RoI regions, they have to go through complex steps such as histogram threshold processing from multiple color space and merging of several binary images. The subsequent functions are based on this binary mask map, such as outputting clustering contours, circumscribed shapes of plant regions and color analysis, etc. The dependence on manual features and parameters reduces their stability and ease of use. Among them, PlantCV can use the Skimage library to skeletonize the mask map, then determine the branch points and tip points of the crop, and use these to finally determine the number of leaves. PlantCV also supports a naive bayes machine learning method. After labeling data and training models, it can achieve multi-classification of pixels with different color performance in crop images, replacing the process of manually setting color thresholds. By contrast, the commercial image analysis software equipped with the lemnatec high-throughput phenotyping system is more mature and complete. It integrates some deep learning methods and can monitor the phenotypic characteristics of specific species in a limited growth cycle. They can identify the shoots, roots, and root hairs of seedlings grown on petri dishes or substrates. They can identify the embryos and endosperms of maize seeds that are neatly arranged, and can also detect emerging cotyledons during oilseed rape germination.

With the rapid development of computer vision and deep learning in recent years, there are more advanced research methods to extract and process visual information of image data. CNN enjoys a stupendous success in object classification, localization, detection, and segmentation. It has been applied on a large scale in the fields of automatic driving, face recognition and remote sensing images analysis, greatly boosting productivity in these areas and achieving huge economic benefits. It is very valuable to explore the powerful capabilities of the CNN-based deep learning methods in image processing and understanding in the domain of plant and agricultural science. Along with the tide of artificial intelligence and deep learning, researchers in the fields of computer vision and plant agriculture began to penetrate both sides. A deep-learning-based convolutional neural network (CNN) and Long Short Term Memory (LSTM) framework aiming at plant classification is proposed and shows its benefits over hand-crafted image analysis [17]. To combat illegal logging, a series of CNN classification models are presented to identify the woods of 10 species in [18]. Based on CNN, a pipeline to detect regions containing flowering panicles and estimate heading date of paddy rice is introduced in [19].

While plenty solutions have met the need of research customized for some specific phenotype and targeted at limited crop species, most of them are either based on digit image processing idea where requires various algorithms with multiple stages to complete the processing, or based on CNN but not taking maize as the research object. To our knowledge, there is no free and easy-to-use image analysis software with GUI for maize sheath point detection and leaf instance segmentation in research community. In actual maize research, a powerful tool that can extract the phenotypic characteristics of interest end-to-end and automatically will undoubtedly greatly improve the efficiency of experiments and provide great help to the plant research community. The software platform proposed in this paper integrates the most popular methods of deep learning and computer vision and implements a variety of phenotypic analysis applications. By using deep learning method, the detection of maize leaf sheath points and the segmentation of leaf instances provided by this software are unprecedented new function among current plant phenotypic image processing software, which can inspire researchers with new study materials and indexes. At the same time, the software supports batch processing, making automatic processing and information extraction of a large number of maize images collected by high-throughput phenotype platforms possible.

Taking the RGB image of maize as input, Maize-IAS can extract the plant area and calculate the number of pixels in the projected area. Then the color analysis is performed on this area: the mean and standard deviation of the three-channel color values are calculated and the color histogram is drawn and displayed. The software can detect and locate the leaf sheath points of maize plant in the image and gives the distance between every two adjacent leaf sheath points (i.e., the stem node). On this basis, the height and stem diameter of the plants can also be calculated. At the same time, Maize-IAS can implement pixel-level instance segmentation of maize leaves, through which the number of leaves is counted. On the test-set, the mean of the difference between the ground truth of leaves number and the inference result reaches down to 1.60, and the standard deviation 1.625. All of the above functions for processing a single image can be completed in real time after clicking the corresponding button. The software also supports batch processing to extract and analyze phenotypic features respectively at an average speed of 100 ms per image (2454 * 2056, using NVIDIA Gefore RTX 2070), allowing users to choose the path where to save the processed results.

In this paper, algorithmic ideas for implementing each part of software function are introduced in section Implementation. The data annotation details, processing results of each function, and the software interface are described in section “Results and discussion”. Finally, section “Conclusion” summarizes this paper and looks forward to the research prospects.

## Implementation

**Fig. 1:**
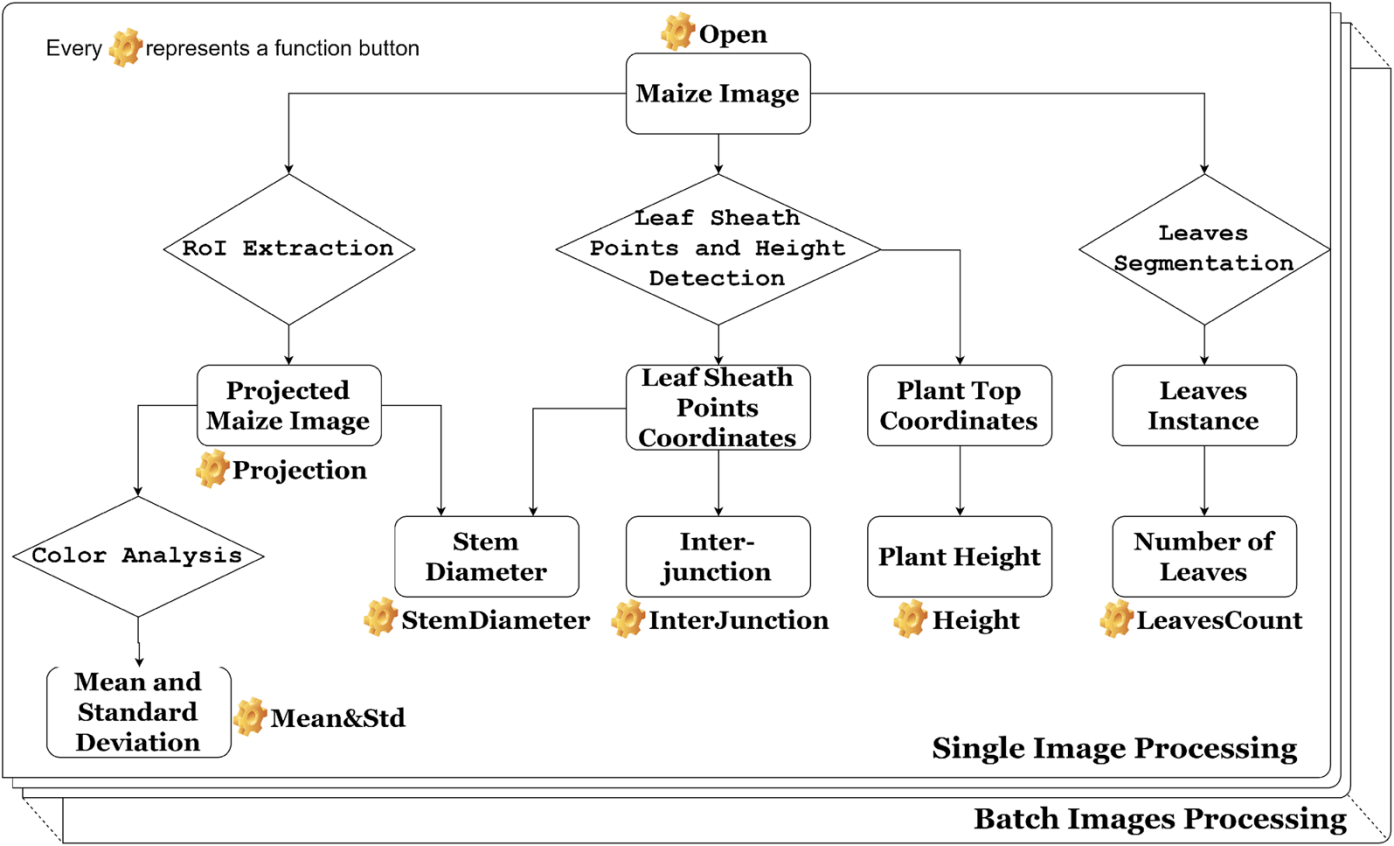
Software function flow chart

Maize-IAS is a software with PyQt5 graphical interface written with Python3.6, which runs on Linux platform only so far. It requires multiple scientific and numeric libraries so it is recommended for users to install an Anaconda Python distribution in operating environment. In addition, the machine will also needs to install the OpenCV Python library (v4.1.1), pytorch (v1.2.0) and torchvision (v0.4.0) deep learning framework. In order to improve the portability of the software and the convenience of installation, we use Pyinstaller to package the software as an executable program under the Linux system. This executable program can be run directly in the terminal after decompression. Way to download the software is introduced in the project home page (see “Availability and requirements” section) and related installation and debug guidelines is provided in (Additional file 1). The software is designed to accept RGB images of maize as input, which kind of image data can be provided by generally all high-throughput plant phenotypic platforms. All calculated numerical results can be generated into “.txt” files in the batch processing function. The whole software function flow chart is shown in Fig.  1.

In this paper, we set the coordinate system of the image as follows: the upper left corner of the image is the coordinate origin, from the origin to the right is the x-axis direction, and the downward direction is the y-axis direction (Fig.  4).

### RoI extraction and color analysis

**Fig. 2:**
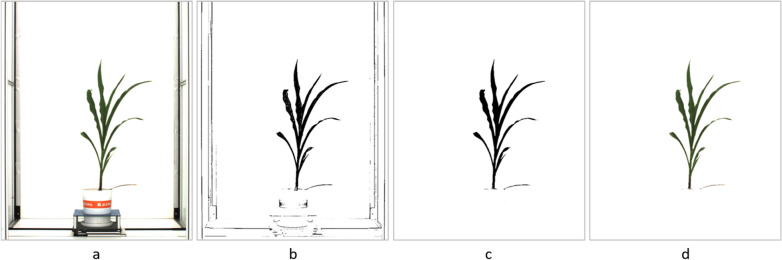
Maize RoI extraction (level view). **a** Original image. **b** Preliminary binary mask image. **c** Optimized mask image. **d** Foreground RGB image

**Fig. 3:**
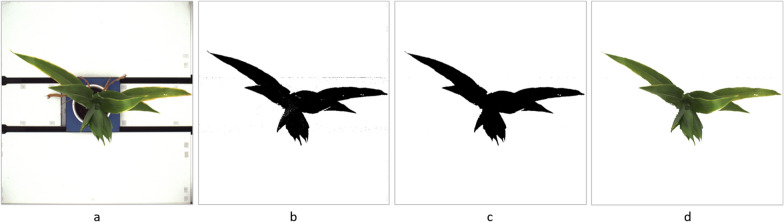
Maize RoI extraction (top view). **a** Original image. **b** Preliminary binary mask image. **c** Optimized mask image. **d** Foreground RGB image

In this study, the region of interest is the maize body in the image (Figs.  2a and  3a), excluding the background board of the image capture chamber, cultivation pot and its fixture to the assembly line. RoI extraction is a basic procedure for most of following steps of phenotypic analysis of image-based data. Considering that the images captured by high throughput plant platforms have an extremely clean and identical background, whose color is very different from foreground objects, we apply color recognition methods to extract the plant body from the background.

The first step is to determine the range of values for each color channel of the foreground object. The original RGB image has a color space designed for machine, whose color numerical value has no approximate linear relationship with color representation. Consequently, it will be very confusing and inaccurate to determine the range of three-channel value separately. Instead, we convert the RGB image into HSV (hue, saturation, value) color space, which aligns more closely with the way human vision perceives color-making attributes. In HSV color space, the pixels value delimitation of the maize body is easy and intuitive to operate. These boundary values are used as a threshold to determine the pixel that is set to 1 (maize body area) or 0 (background area) in the image binarization course. Consequently, we can get a preliminary binary mask image (Figs.  2b and  3b) of the maize plant. In order to optimize extraction result, the binary mask will be subjected to morphological operations, including erosion processing to remove noisy pixels at the area out of maize body, and dilation processing to fill the tiny holes inside the plant body (Figs.  2c and  3c). Then we align the binary mask image with the original image to produce RGB color image with maize body only (Figs.  2d and  3d).

After getting the masked RGB image of the plant body, we have convenient access to implement various processing only on the pixels of RoI. In this function page, we count the total number of RoI pixels. Combined with camera parameters and environment settings, the real projected area of maize body in the image and its true size can be calculated. Color analysis about mean and standard deviation of three channels of RoI is performed as Eqs. (1) and (2). Here *c* represents 3 color channel of RGB and $p_{c,(i, j)}$ is the value of pixel(i, j) of color channel *c*. The sum from $(h_{0}, w_{0})$ to $(h_{n}, w_{n})$ represents the accumulation of foreground pixels of the extracted maize plant and $num_{pixel}$ is the total number of pixels in the RoI. Its color histogram is drawn.

1 $$\begin{aligned} {mean}_{c}= & {} \left[ \sum _{(i, j)=\left( h_{0}, w_{0}\right) }^{(i, j)=\left( h_{n}, w_{n}\right) } p_{c,(i, j)}\right] / num_{pixel} \end{aligned}$$

2 $$\begin{aligned} std_{c}= & {} \sqrt{\left[ \sum _{(i, j)=\left( h_{0}, w_{0}\right) }^{(i, j)=\left( h_{n}, w_{n}\right) }\left( p_{c,(i, j)}-mean_{c}\right) ^{2}\right] / num_{pixel}} \end{aligned}$$

### Leaf sheath points and height detection

The Stem Node is the node where a leaf grows out of the stem and the Internode Length is the distance between two adjacent nodes, which is the distance between two adjacent leaf sheath points. So the problem of measuring the internode length can be transformed into the problem of detecting the leaf sheath points. The current object detection algorithm has a good performance on the mainstream benchmark dataset like PASCAL VOC [20]. But the maize dataset generally has a character of much higher resolution and relatively smaller objects to be detected. When applying the common object detection algorithm like Faster R-CNN [21] to the maize dataset, the original image needs to be downsampled due to GPU memory limitations. But this will in turn results in the loss of detailed information describing the characteristics of the small object, like location.

In order to solve this contradiction, in our previous work [22] a Small Object Detection method guided by prior knowledge from coarse to fine is proposed. In the task of detecting leaf sheath points, the target image is highly structured. That is, the position of the leaf sheath point is likely to appear in the center of the image, rather than at the edge of the image. Such prior knowledge can be used as a constraint to guide the detection process. To obtain prior knowledge, the probability map of the position of leaf sheath point is computed from labeled training image, then expanded, eroded and blurred. To achieve high precision, the concept of two-stage detection in Faster R-CNN methods is borrowed. In the first phase, an area that may contain the objects is roughly find out, namely the leaf sheath region. The original image is downsampled to an appropriate size to reduce the burden of GPU memory. In the downsampled image, feature maps of different scale are calculated through the backbone network (ResNet50 [23] with FPN [24]). Then, prior knowledge is used to guide the RPN network to generate RoIs. These RoIs are rough and need to be further classified. In the second stage, the features of these small RoIs are calculated from the high-resolution image for fine classification. This method makes the detection accuracy of the leaf sheath point higher.

**Fig. 4:**
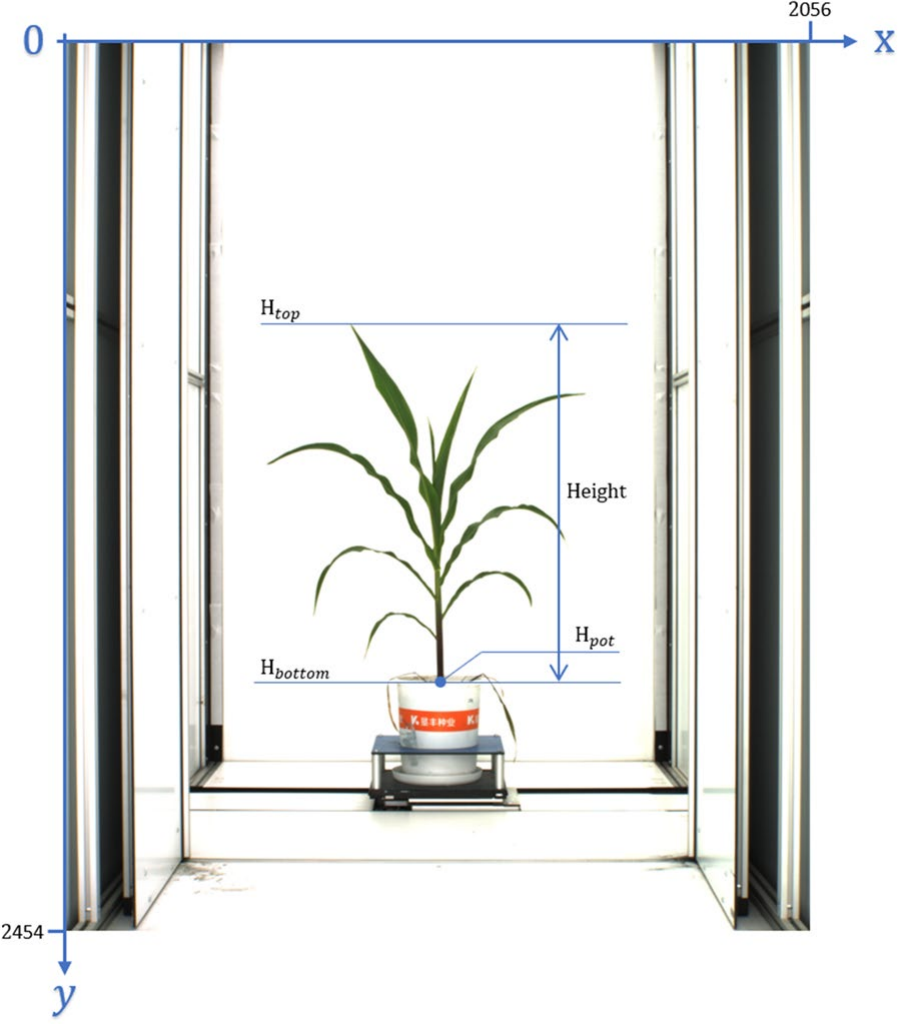
Height definition and coordinate system

Along with the leaf sheath points detection, the end tip of the leaf at the most top position of the image (H$_{top}$) is detected, as well as the most bottom position of maize stem (H$_{bottom}$). Plant height is defined as the distance from the soil surface to the farthest end of the plant in the direction of plant growth, which is the distance between the above two points (Fig.  4).

### Stem diameter

**Fig. 5:**
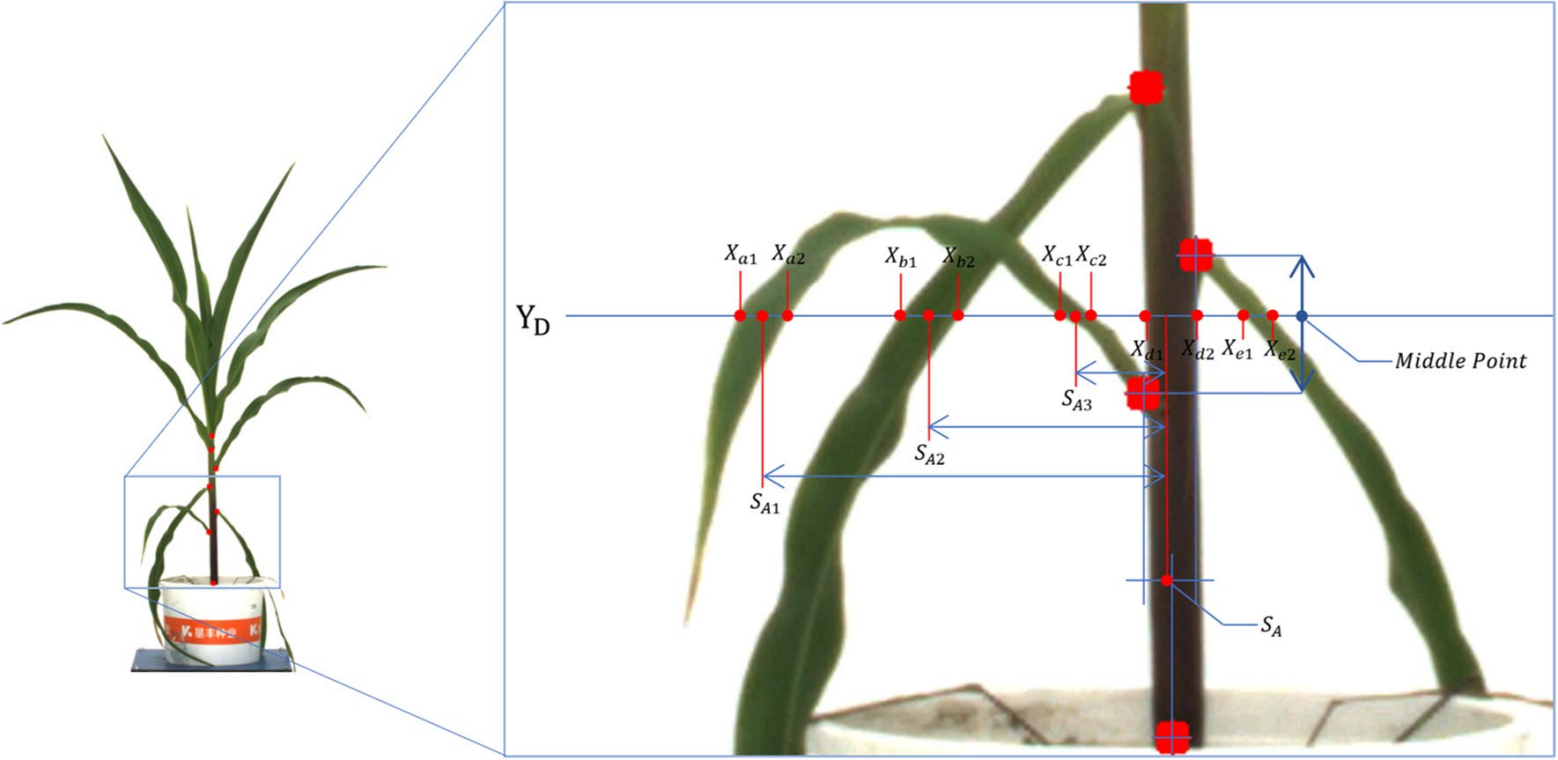
Stem diameter definition

Stem diameter here is defined as the cross-sectional diameter of the middle of the second stalk from the soil surface. Since we have already got the binary mask image in the first section and all the junction coordinates in the second section, obviously the second stalk is between the second junction and the third junction from the soil surface. Now we can easily determine the horizontal position (y-axis = Y$_D$) where to measure the stem diameter. Firstly, in the binary mask image, traverse all the pixels on this Y$_D$ horizontal line in order and determine the coordinates where pixel value changes from 0 to 1 and from 1 to 0. These coordinates ((X$_{a1}$,Y$_D$),(X$_{a2}$,Y$_D$); (X$_{b1}$,Y$_D$),(X$_{b2}$,Y$_D$);...) are the intersection between the edge of leaves&stem and the horizontal line. All these coordinates are paired and between every two adjacent of them are leaf or stem pixels. Secondly, select the two junction nodes with the largest y-axis coordinate (except the H$_{pot}$ point) and calculate the average value of their x-axis coordinates. The average value, named as S$_A$, is excepted to be the approximate x-axis range where the stem is located in the image. Finally, compare the distance between the midpoint of all point pairs (S$_{A1}$ = (X$_{a1}$+X$_{a2}$)/2, S$_{A2}$, etc.) and S$_A$, choose the smallest one, and the distance between these two endpoints is the stem diameter, as shown in Fig.  5.

### Leaves counting

Leaves counting is a more challenging task. In [25], thanks to the radial growth pattern of Arabidopsis, the distance between the arabidopsis plant centroid and its leaf contour (at angles from 0 and 360 degrees with a 15-degree interval) is used as a criterion to judge whether there is a leaf. In more related work of [26], maize leaves number are counted after the architecture determination operating, including extraction, skeletonization and complicated graphical representation of a plant, based on digital image processing methods. Here not only do we require to know the number of leaves of the maize plant in an image, but we also extract the edge contours of every single leaf to obtain the individual mask for each leaf, which is actually an instance segmentation task. Given that our custom dataset is small, we follow the approach of Mask R-CNN [27] framework, and fine-tune an instance segmentation model pre-trained on COCO dataset [28]. Faster R-CNN [21] is real-time object detection network with branches for classification and bounding box regression, which can output a rectangular box wrapping object of a specific category. FCN [29] can perform semantic segmentation on images which is pixel-to-pixel multi-classification of images.And Mask R-CNN is an extension of Faster R-CNN by adding a branch of a small FCN for predicting segmentation masks on each RoI, so that it can output mask for every object in every category.

We conduct transfer learning on the Mask R-CNN, which is based on top of Faster R-CNN backbone. To fine-tune the backbone network for predicting the domain-specific classes, we replace the pre-trained head classifier with a new FastRCNNPredictor that has the number of classes defined by our task. Here the number of classes is simply set as 2, representing two categories of foreground maize body and background. Because we also need to compute the instance segmentation masks, so a new MaskRCNNPredictor of RoI heads with compatible input features number is also replaced. Before feeding image data to the network, randomly flip the training images for data augmentation.

Function of leaf sheath point detection and leaves counting are not related to maize’s growth stage, and there is no upper or lower limit to number of leaves. As long as they match the feature of leaves and leaf sheath points, they will be segmented and detected.

## Results and discussion

The maize image dataset used in the software test is from the Institute of Crop Sciences, Chinese Academy of Agricultural Sciences. Images were collected using the high-throughput plant phenotyping platform system Lemnatec Scanalyzer 3D. During the growing period of maize, images were captured every three days from the seedling stage to the filling stage, and were taken from three angles of ${0}^{\circ }$, ${90}^{\circ }$ in the horizontal direction and the direction of the top of the maize. The resolution size of the image is 2454 * 2056. The maize dataset is labeled using two different annotation methods to construct two sub-datasets, which correspond to detection and segmentation problems respectively.

The Maize-IAS application supports fast one-click analysis and its use is simple and clear.

### Projection and color analysis

**Fig. 6:**
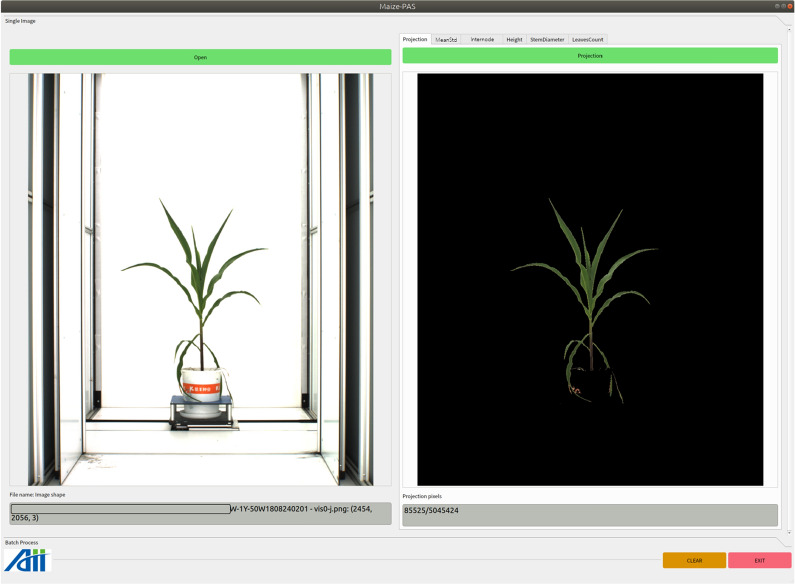
Functional interface for calculating projected area of plant area

**Fig. 7:**
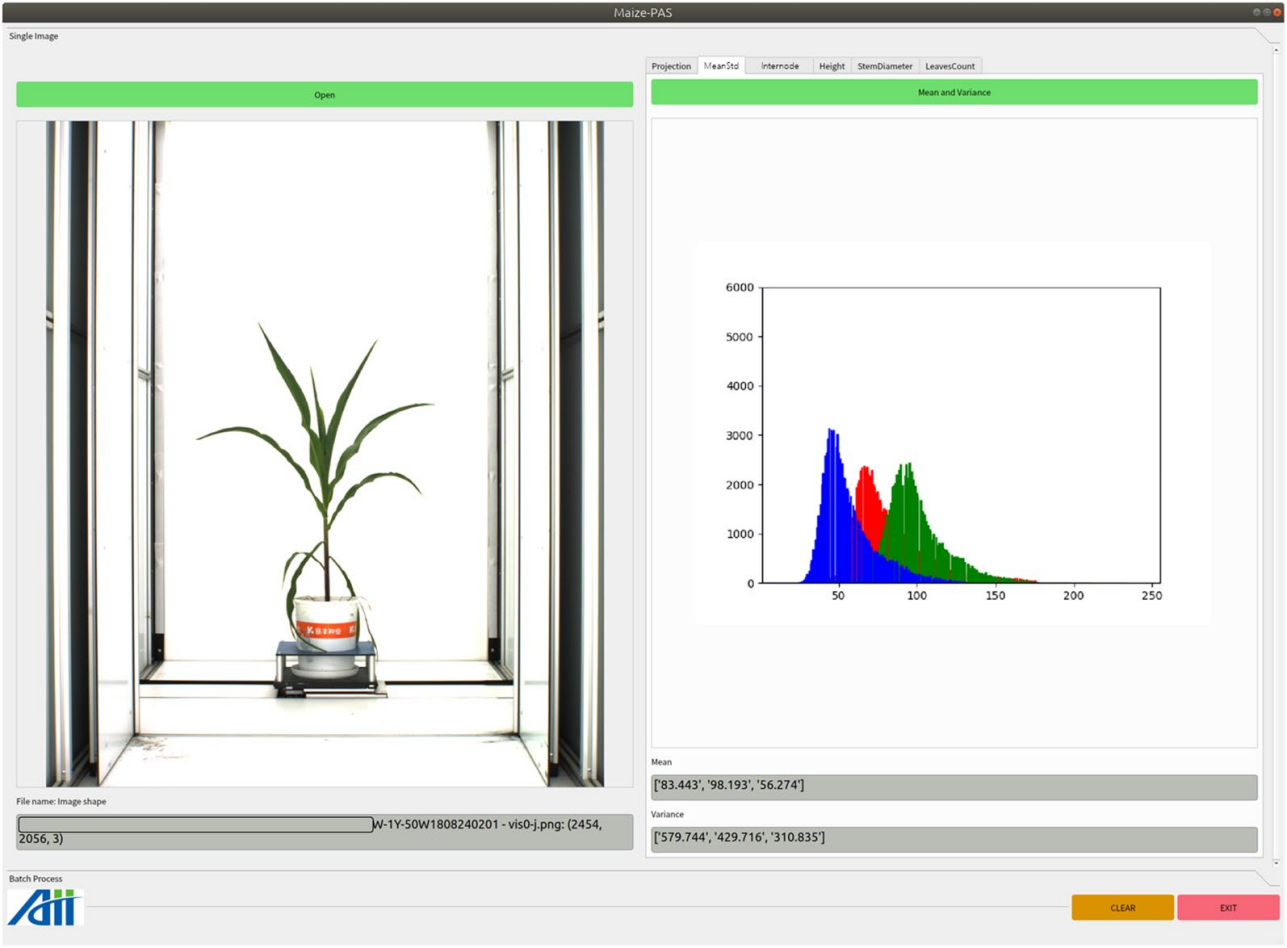
Functional interface for calculating the mean and standard deviation of the three channel colors in the plant area

Click the pushbutton *Open* to load in a maize image. The original image will be displayed. It’s path, file name and pixel shape will be listed below. Click the pushbutton *Projection* to start the processing, then the projected RGB image will be displayed. The total number of maize area pixels and original image pixels will be listed, as shown in Fig.  6. Click the pushbutton *MeanStd* to start calculating the mean and standard deviation of the maize area in the image, result and histogram will be output, as shown in Fig.  7.

For the dataset used in this study, the RoI extraction method based on color recognition is sufficient to obtain accurate results. In the case study of segmenting maize leaves, with the increase of labeled data fed into the deep neural network, the segmentation effect will grow better, and the edge of the mask will become sharper. Further discussion is given below.

### Internode length and height detection

**Fig. 8:**
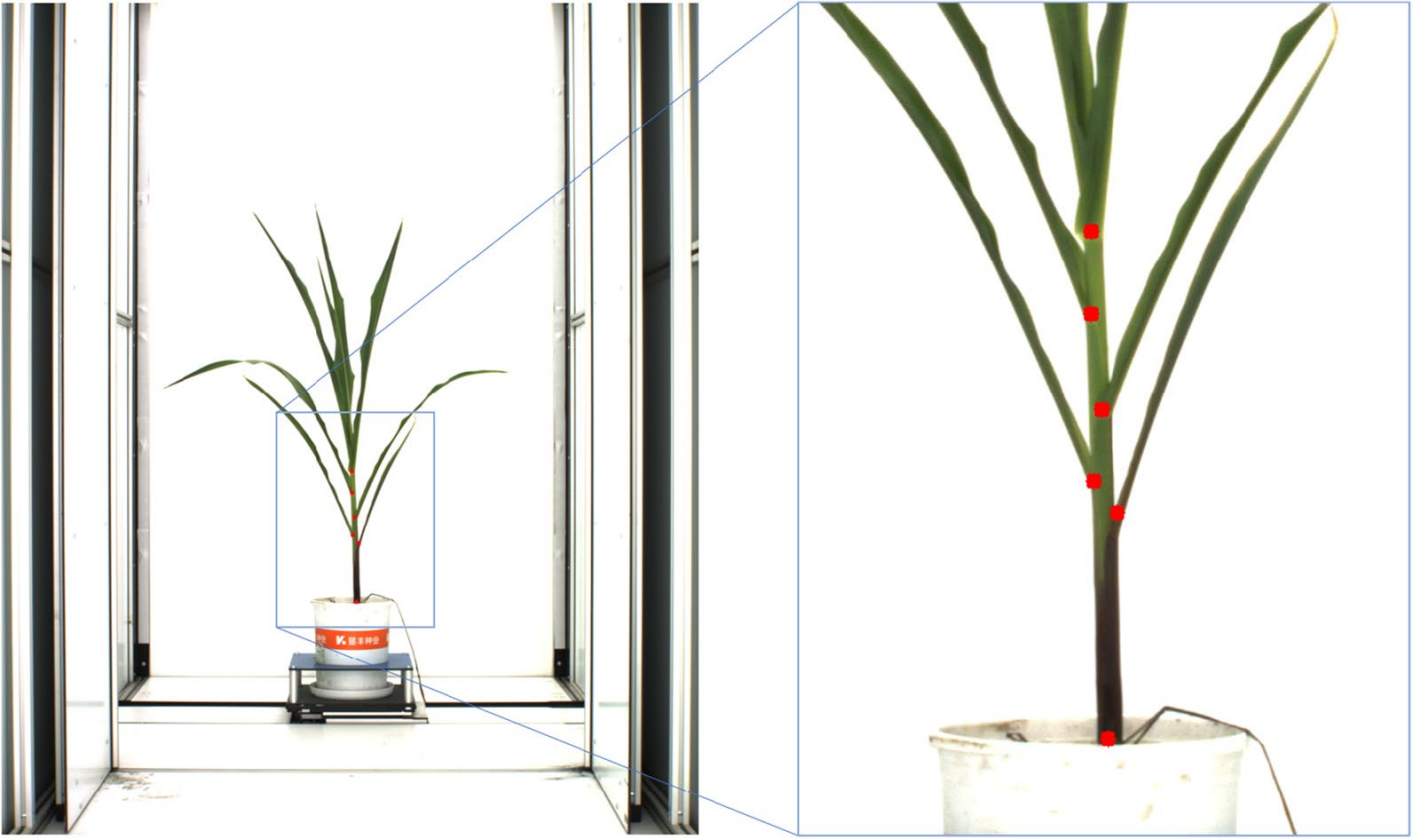
Leaf sheath points labeled on maize image

To train the deep network, images labeled with ground truth are essential. We create a labeled maize dataset consisting of 520 maize images, where 370 of them are used as the training set and 150 as the test set. Leaf sheath points of the maize plant in these images are manually labeled by professional researchers in the agriculture field, as shown in Fig.  8.

**Fig. 9:**
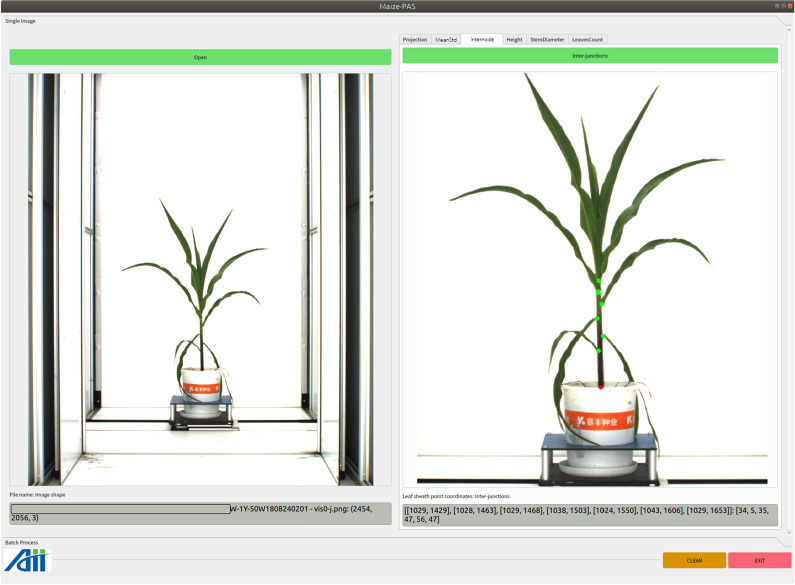
Functional interface for detecting the internode length

**Fig. 10:**
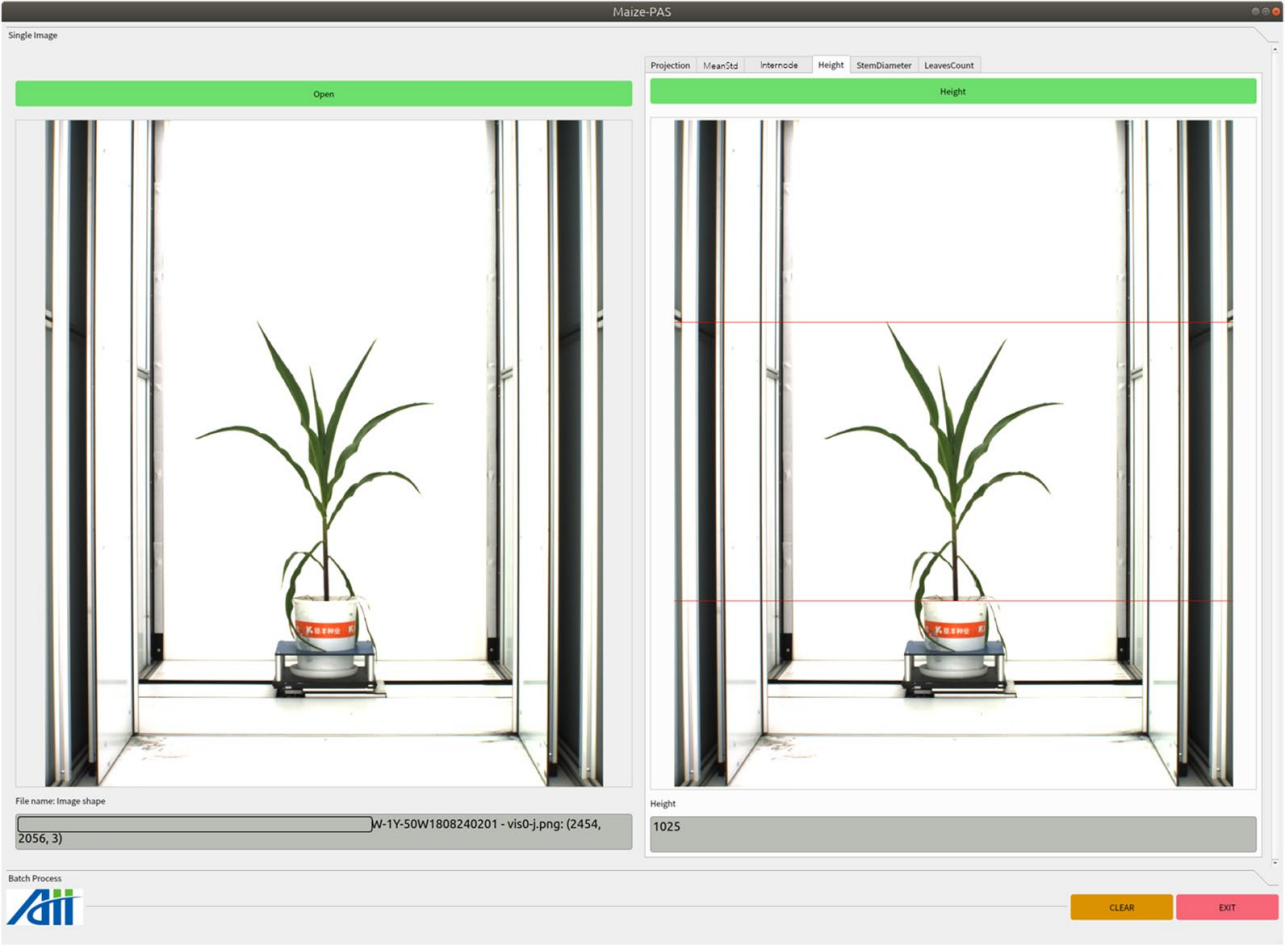
Functional interface for detecting the maize plant height

Click the pushbutton *Open* to load a maize image, the original image will be display below. Cilck the pushbutton *Internode* to initiate the detection, and the visualization result is the ouput, as shown in Fig.  9. Click the pushbutton *Height* to detect the plant height, with both ends of maize plant are marked with horizontal lines, as shown in Fig.  10.

When a maize image of a particular viewing angle is detected, defects of inability to detect all leaf sheath points caused by occlusion between the leaves are inevitable. Further research can fuse image information from different perspectives, and finally accurately detect the leaf sheath points of the maize plant.

### Stem diameter

**Fig. 11:**
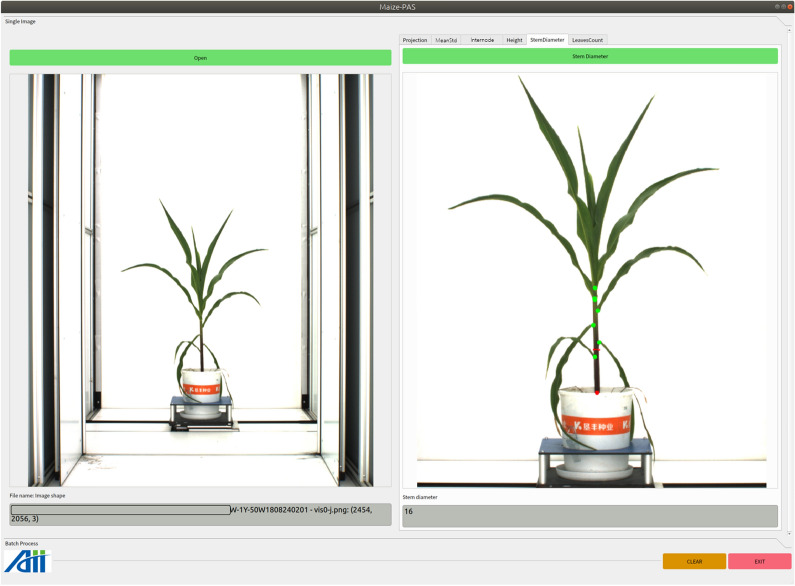
Functional interface for detecting the maize stem diameter

Click the pushbutton *Open* to load a maize image, the original image will be display below. Cilck the pushbutton *StemDiameter* to detect the stem radius with red lines as the measuring position. The diameter measuring position of the mazie stem is marked by a short red line and the diameter of the stem in pixels is displayed below, as shown in Fig.  11.

### Leaves counting

**Fig. 12:**
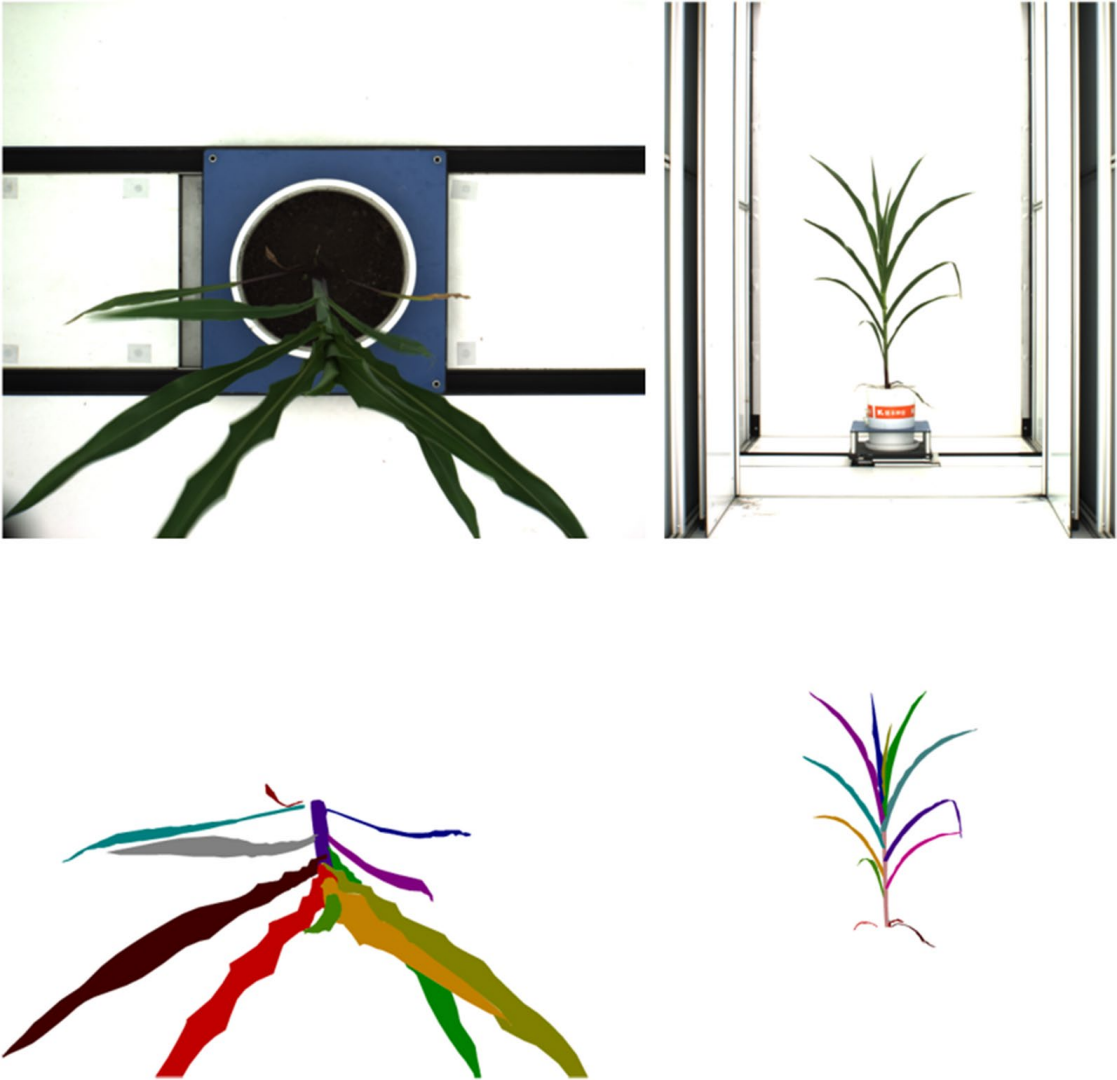
Leaves segmentation label sample

we build a custom maize dataset to train the CNN model. The segmentation task is pixel-level classification, which means that our dataset also demands pixel-wise annotations. We use the open-source annotation tool Labelme [30] (Image Polygonal Annotation with Python) to make polygonal annotation on original maize images. In the course of manual labeling, every piece of leaf and the main stem are surrounded by a polygon with its real categories attached. Category labels include leaf, stem, and background. Among them the label *background* is split automatically by Labelme application. The exampled labeled images from different views are shown in Fig.  12. We create a dataset consisting of 253 maize images, among them, 172 are siding view images at ${0}^{\circ }$ and ${90}^{\circ }$, and the remaining 81 are top view images. In statistically, there are approximately 10 different leaf masks in every labeled maize image.

**Fig. 13:**
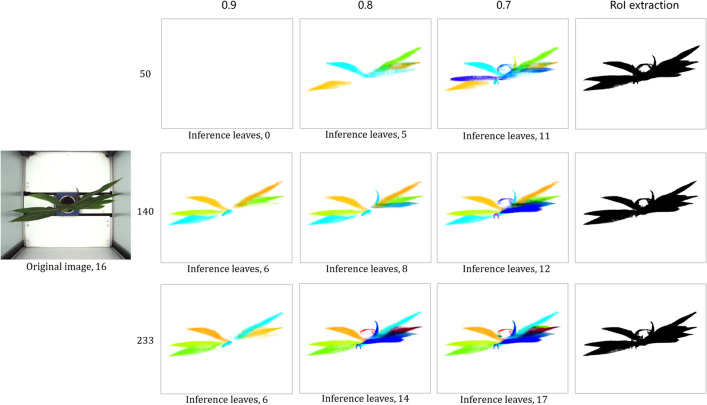
Case of top view. The three rows from top to bottom are the inference results with the training set sizes of 50, 140 and 233. Each row from left to right is the result of leaves inference at confidence of 0.9, 0.8, 0.7 and roI extraction respectively

**Fig. 14:**
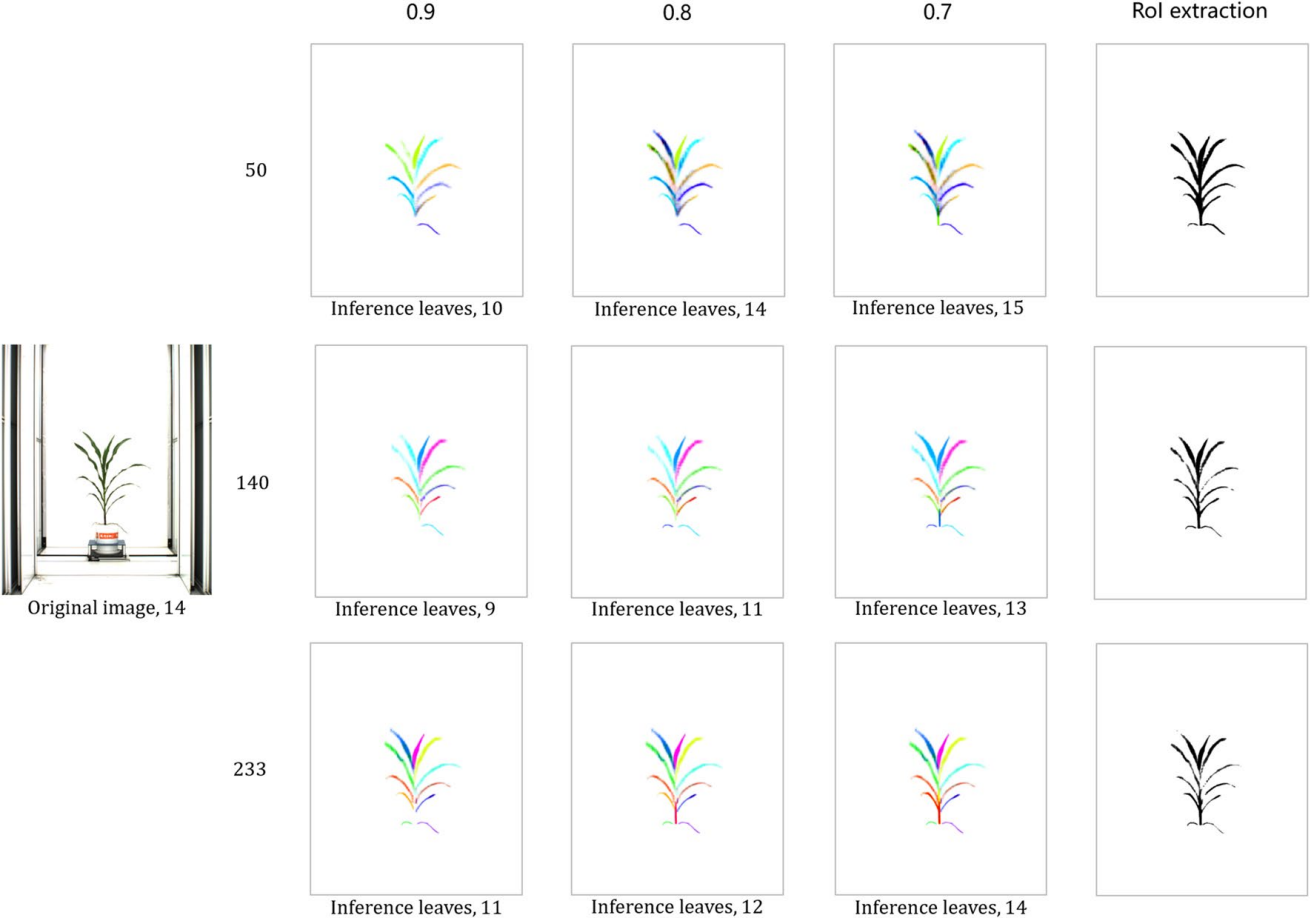
Case of level view. The three rows from top to bottom are the inference results with the training set sizes of 50, 140 and 233. Each row from left to right is the result of leaves inference at confidence level of 0.9, 0.8, 0.7 and roI extraction respectively

We conduct experiments with different amounts of training data. After feeding the network 50, 140 and 233 training images separately, the prediction results are shown in Figs.  13 and  14. As the amount of training data increases, the prediction result is obviously growing better and the statistical results of the number of leaves are more accurate with the same confidence. The corresponding mean and standard deviation of the difference between the inference value and the ground truth value are shown in Table  1. The algorithm performs best with a confidence of 0.7.

**Table 1:** Mean and standard deviation of difference between the inference and ground truth

Confidence	0.9	0.8	0.7	0.6	0.5
Mean	4.90	2.95	1.60	2.60	4.85
Std	3.239	2.729	1.625	1.818	3.198

**Fig. 15:**
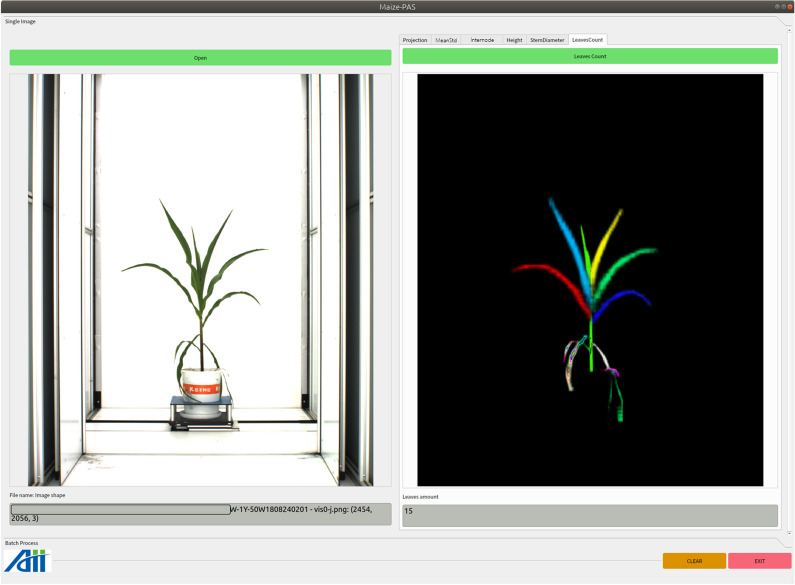
Functional interface for leaves counting

Click the pushbutton *Open* to load a maize image, the original image will be display below. Click the pushbutton *LeavesCount* to segment leaves, and different color and position represent different leaf instance, as shown in Fig.  15.

### Batch processing

**Fig. 16:**
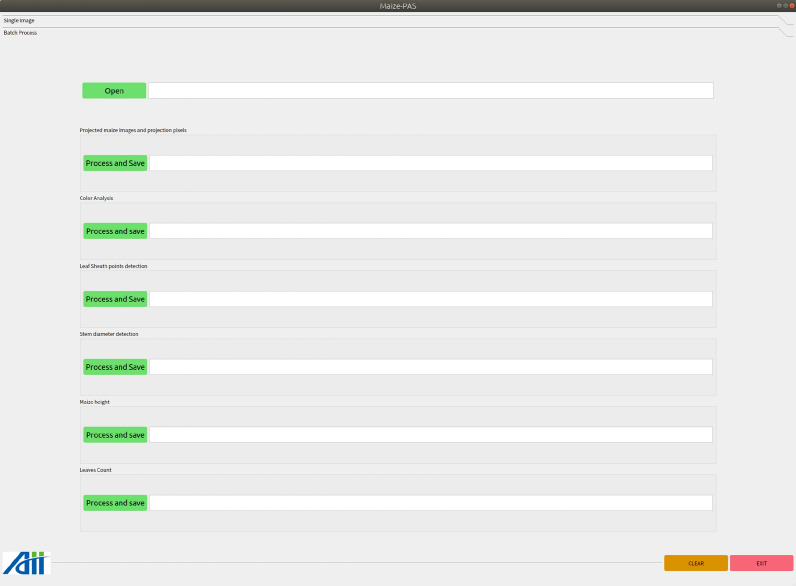
Functional interface of batch processing

In batch processing, click the *Open* button to select the folder where the image collection is located, then click the *Process and Save* button to start the related processing to the image set and store the result file in the path where the original collection is located. Click *Open and Process* to directly select the images’ path and perform the operation, as shown in Fig.  16.

**Fig. 17:**
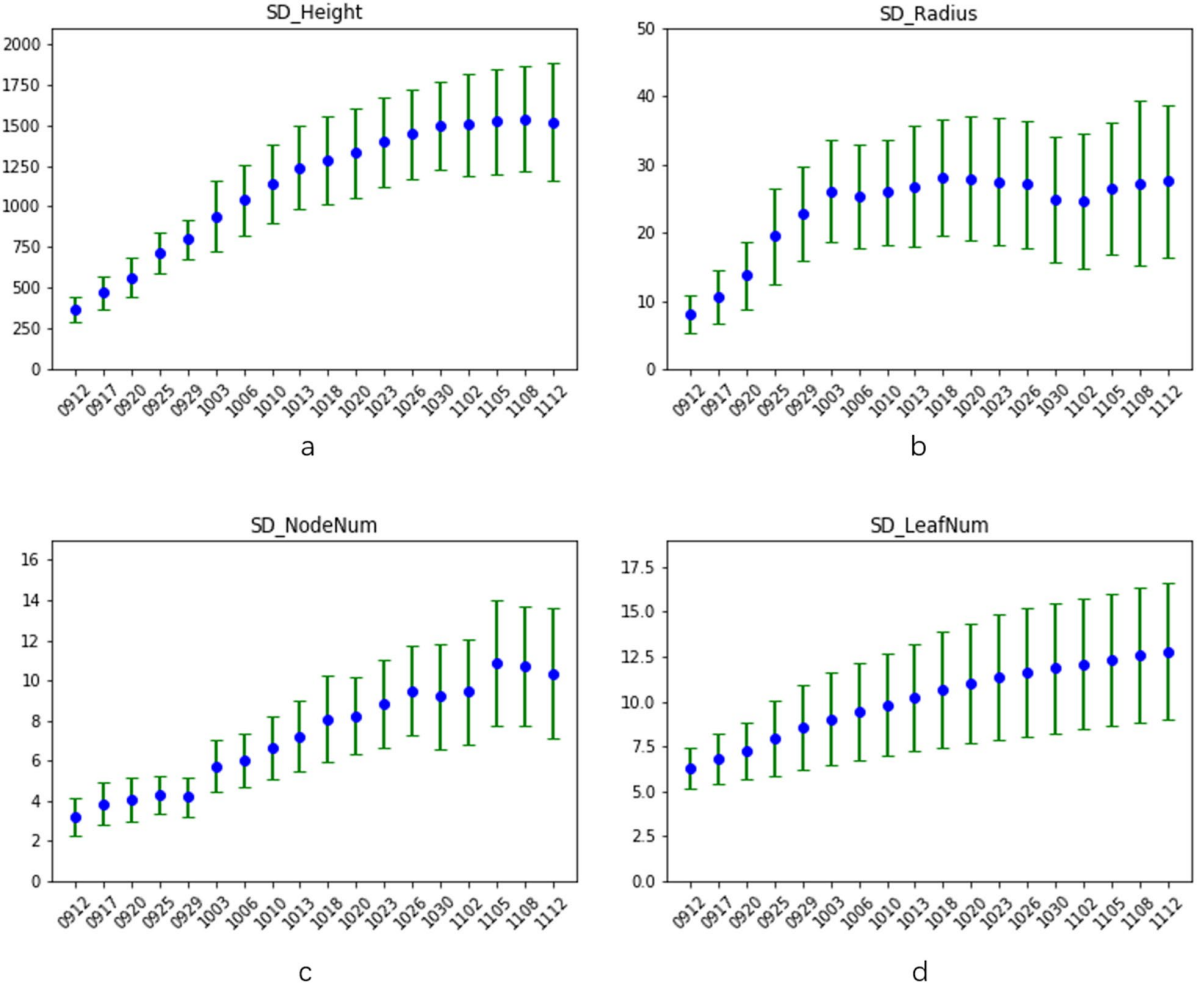
Standard deviation plot of maize changing over time. **a** SD plot of height changing over time. **b** SD plot of stem radius changing over time. **c** SD plot of number of leaf sheath points changing over time. **d** SD plot of number of leaves changing over time

**Fig. 18:**
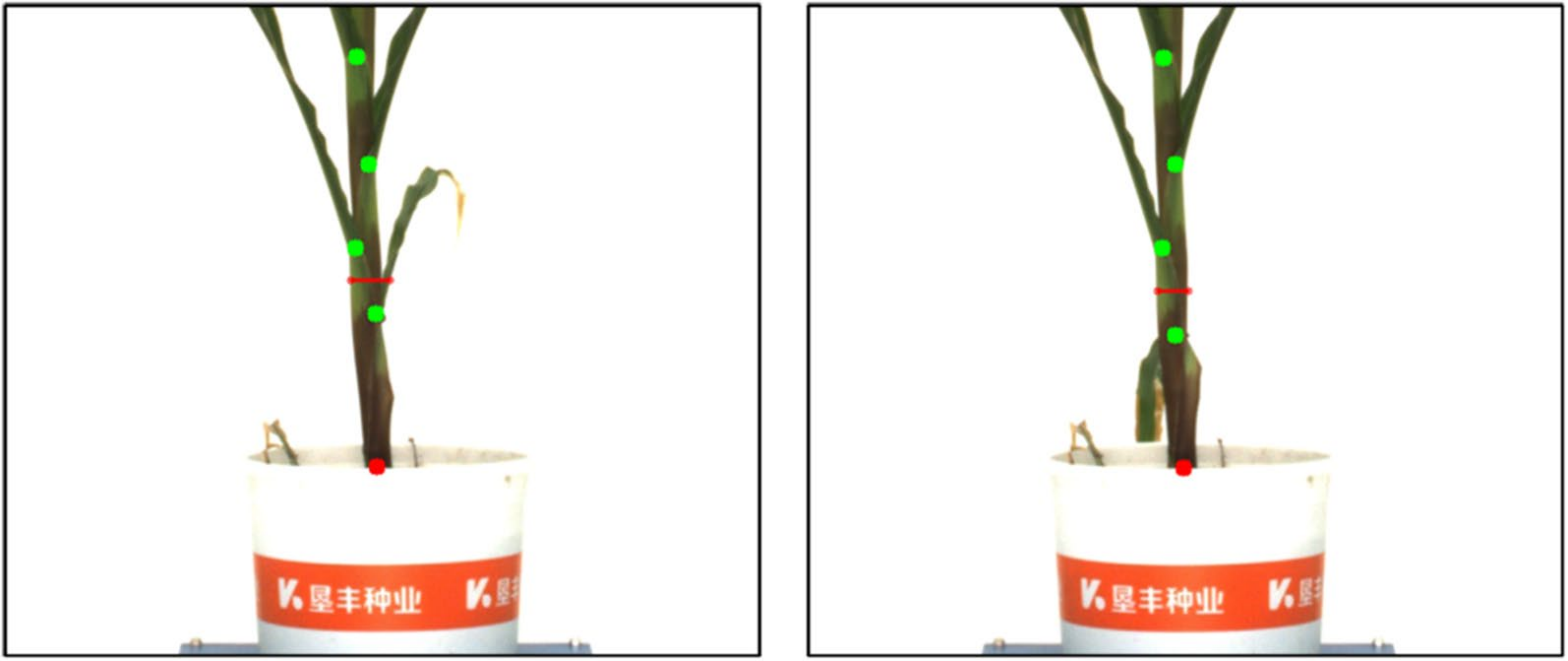
The stem radius decrease because leaves originally wrapped at the bottom of the main stem stretched out (left: 1026, right: 1030)

**Fig. 19:**
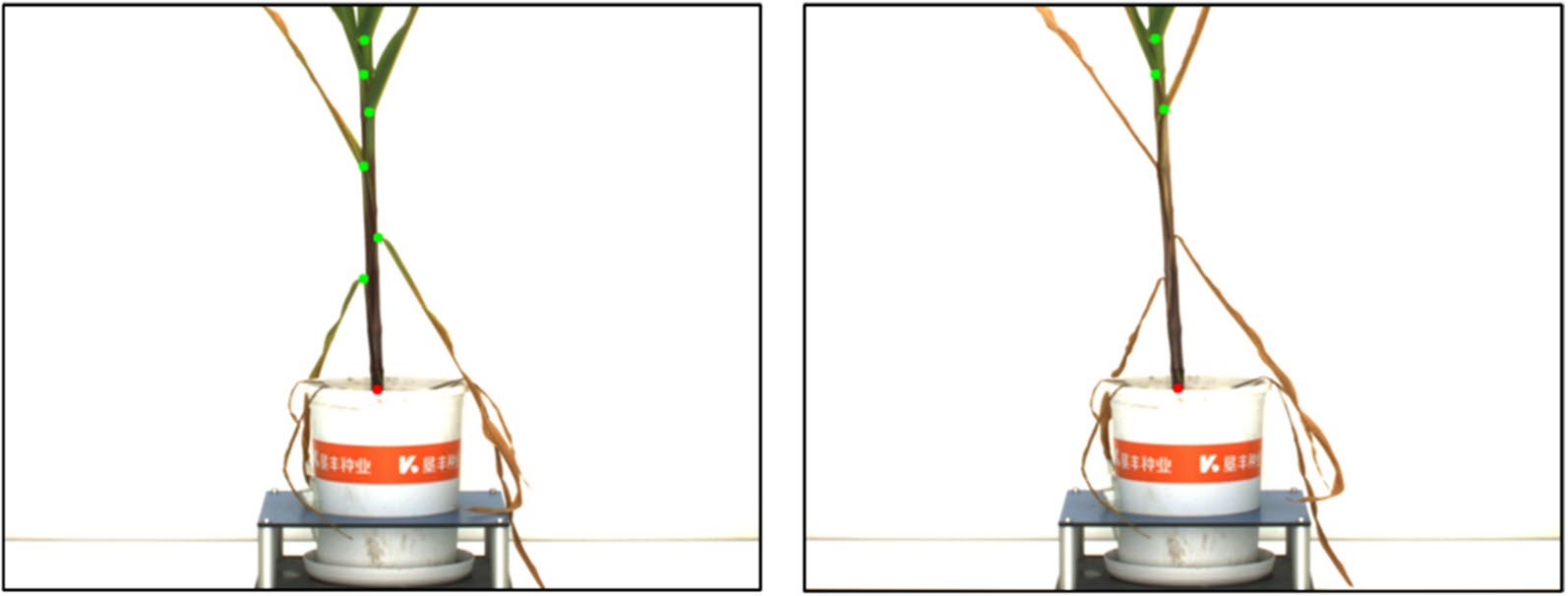
The feature where leaves wither is not enough for the model to recognize them as leaf sheath points (left: 1026, right: 1030)

From the maize data set collected for 18 consecutive months, we randomly select 5 images each month, and apply the batch processing function to these 90 images. The trend of plant height, stem diameter, number of leaf sheath points and number of leaves changing over time is shown in Fig.  17. The abscissa is the date, and the ordinate is the number of pixels or 1. We can observe the trend of the continuous growth of maize plant height and number of leaves from subgraphs a and d. In the subgraphs of stem radius and number of leaf sheath points, the data from 1026 to 1030 showed a downward trend. The former was due to the fact that at stage 1030 and 1102, some leaves originally wrapped around the bottom of the main stem stretched out, resulting in a decline in stem radius as shown in Fig.  18. The latter is because at that growth stage, the leaves at the bottom of maize stem began to wither or even fall. The feature of the normal leaf sheath points became less obvious. Plus there are few similar images in the data set, so the model does not recognize them as leaf sheath points, as shown in Fig.  19. This is also the reason why the number of leaf sheath points continued to decline in the last three date.

Then manually label the above four attributes of these 90 images. We perform a statistical analysis on the difference between the software processing result and the ground truth to obtain the mean and standard deviation of the difference, as shown in the Table  2. Here gt_mean is the mean value of ground truth, dif_mean is mean of the difference between ground truth and processing results, and dif_std is standard deviation of the difference between ground truth and processing results.

**Table 2:** Mean and standard deviation of difference between the inference and ground truth on 90 images

	Height (/pixels)	Radius (/pixels)	NodeNum (/1)	LeafNum (/1)
gt_mean	1222.233	22.044	6.978	10.689
dif_mean	11.284	2.869	1.233	1.611
dif_std	10.279	3.146	1.265	1.372

## Conclusions

The high-throughput plant phenotypic platform makes it possible to automate the monitoring of large numbers of plants. With the collection of consequent huge amounts of imaged-based data, the problem of how to quickly extract the phenotypic characteristics we require from the results comes forth. This paper explores the possibility of AI empowering agriculture and proposes a software for maize phenotype measurement. Standing in the perspective of agriculture and plant science, a small object detection method based on Faster R-CNN [21] is used to detect the leaf sheath points and a fine-tuned Mask R-CNN model completes the instance segmentation of leaves and stem. Meanwhile, to train the deep neural network, maize images dataset labeled manually with task-specific ground truth is build. Statistical analysis is implemented to evaluate the accuracy and effect of these methods. The Maize-IAS version1.0 integrates advanced technologies in machine vision to automatically solve multiple image-based maize phenotypic analysis tasks, including interndoe length, height, stem diameter, RoI segmentation, color analysis and leaves counting. All of the above phenotype data is widely used to analysis maize growth conditions, more extensive research can be developed upon these data. We reveal the potential development prospects of visual phenotype detection using deep learning methods. The methods and workflow provided in this article can also be easily applied to other crops.

## Availability and requirements

Project name: A Maize Image Analysis Software using Deep Learning for High-throughput Plant Phenotyping.

Project home page: https://github.com/surefyyq/Maize-IAS

Operating system: Ubuntu18.04.

Programming language: Python3.

Other requirements: Pytorch 1.1.0 or higher, Torchvision 0.3.0 or higher.

Any restrictions to use by non-academic: None.

## Supplementary information

**Additional file 1.** Installation and debug guidelines.

## Data Availability

The datasets generated and analysed during the current study are not publicly available due academic confidentiality but are available from the corresponding author on reasonable request.
