# Supplementary material for: Maize-IAS: a maize image analysis software using deep learning for high-throughput plant phenotyping
Source: Plant Methods. 2021 Apr 29;17:48. doi: 10.1186/s13007-021-00747-0 (PMC8086349; doi:10.1186/s13007-021-00747-0)
Supplement: Supplementary file 1 — Additional file 1. Installation and debug guidelines. [file 13007_2021_747_MOESM1_ESM.pdf]

Maize-IAS Installation and Debug Guidelines

After you download the Dist2.zip file from figshare, a compressed file of the executable programe:

1. Installation

    step1: Unzip the file, go to the main file directory "Dist2".

    step2: Enter "./dist/run8/run8" in the terminal to start the software.

    step3: Use it according to the method described in the paper and instructions in the software.

2. Issues debug
    a. Symbol lookup error

`symbol lookup error:/.../libmkl_intel_thread.so: undefined symbol: __kmpc_global_thread_num`

    Solutions:

    a. Type `echo $LD_LIBRARY_PATH` in terminal to check your LD_LIBRARY_PATH;

    b. Use `locate libmkl_intel_thread.so to find the path of libmkl_intel_thread.so` in your system;

    c. export it to LD_LIBRARY_PATH:

```
export LD_LIBRARY_PATH=/YOUR PATH to libmkl_intel_thread.so/:$LD_LIBRARY_PATH
```
